# Supplementary material for: Infected erythrocytes and plasma proteomics reveal a specific protein signature of severe malaria
Source: EMBO Mol Med. 2024 Jan 31;16(2):6. doi: 10.1038/s44321-023-00010-0 (PMC10897182; doi:10.1038/s44321-023-00010-0)
Supplement: Supplementary file 1 — Appendix [file 44321_2023_10_MOESM1_ESM.pdf]

Appendix : Table of Content

Figure	Name	N° page
Appendix Figure S1	Over-represented biological pathways from plasma proteins exclusively quantified in CM (n=114) assessed by PANTHER GO online tool.	2
Appendix Figure S2	Interaction networks and over-represented pathways from plasma differentially abundant Host proteins assessed by Ingenuity pathways Analysis.	3-4
Appendix Figure S3	Interaction networks and over-represented pathways from iE differentially abundant Host proteins assessed by Ingenuity pathways Analysis.	5-6
Appendix Figure S4	Over-represented biological pathways from iE differentially abundant parasitic proteins assessed by PANTHER GO online tool.	7-8
Appendix Figure S5	Reverse-Phase High pH Chromatography fractionation	9

Appendix Figure S1.

PANTHER GO-Slim Biological Process	Homo sapiens (REF) #	upload_1 (▽ Hierarchy NEW! ⓘ) #	expected	Fold Enrichment	+/-	raw P value	FDR
intra-Golgi vesicle-mediated transport	24	3	.12	24.51	+	3.40E-04	3.14E-02
DNA-dependent DNA replication	61	4	.31	12.86	+	3.34E-04	3.22E-02
↳metabolic process	5907	52	30.12	1.73	+	7.13E-06	1.13E-03
↳organic substance metabolic process	5685	51	28.99	1.76	+	5.40E-06	9.22E-04
↳macromolecule metabolic process	4815	42	24.56	1.71	+	1.78E-04	1.80E-02
↳cellular metabolic process	5332	47	27.19	1.73	+	2.97E-05	3.88E-03
mRNA splicing, via spliceosome	176	9	.90	10.03	+	4.31E-07	1.37E-04
↳mRNA processing	229	11	1.17	9.42	+	3.88E-08	4.31E-05
↳RNA processing	481	15	2.45	6.11	+	3.09E-08	6.85E-05
↳RNA metabolic process	2410	25	12.29	2.03	+	6.21E-04	4.60E-02
↳nucleic acid metabolic process	2686	30	13.70	2.19	+	2.83E-05	3.93E-03
↳nucleobase-containing compound metabolic process	2928	34	14.93	2.28	+	2.13E-06	4.31E-04
↳cellular nitrogen compound metabolic process	3265	40	16.65	2.40	+	4.56E-08	3.37E-05
↳nitrogen compound metabolic process	5018	46	25.59	1.80	+	1.76E-05	2.61E-03
↳organic cyclic compound metabolic process	3041	36	15.51	2.32	+	5.95E-07	1.32E-04
↳heterocycle metabolic process	2978	36	15.19	2.37	+	3.94E-07	1.75E-04
↳primary metabolic process	5251	46	26.78	1.72	+	6.70E-05	8.26E-03
↳cellular aromatic compound metabolic process	2989	36	15.24	2.36	+	4.21E-07	1.56E-04
↳gene expression	2894	30	14.76	2.03	+	1.08E-04	1.26E-02
↳mRNA metabolic process	316	12	1.61	7.45	+	1.07E-07	5.96E-05
↳RNA splicing, via transesterification reactions with bulged adenosine as nucleophile	176	9	.90	10.03	+	4.31E-07	1.20E-04
↳RNA splicing, via transesterification reactions	176	9	.90	10.03	+	4.31E-07	1.06E-04
↳RNA splicing	221	9	1.13	7.99	+	2.64E-06	4.88E-04
small molecule catabolic process	122	5	.62	8.04	+	4.76E-04	4.06E-02
monocarboxylic acid metabolic process	152	6	.78	7.74	+	1.56E-04	1.73E-02
ribonucleoprotein complex biogenesis	223	7	1.14	6.16	+	1.73E-04	1.83E-02
DNA repair	197	6	1.00	5.97	+	5.95E-04	4.55E-02
↳cellular response to DNA damage stimulus	252	7	1.29	5.45	+	3.56E-04	3.16E-02
Unclassified	9598	31	48.95	.63	-	5.34E-04	4.24E-02

Appendix figure S1. Over-represented biological pathways from plasma proteins exclusively quantified in CM (n=114) assessed by PANTHER GO online tool.

Appendix Figure S2.

A.

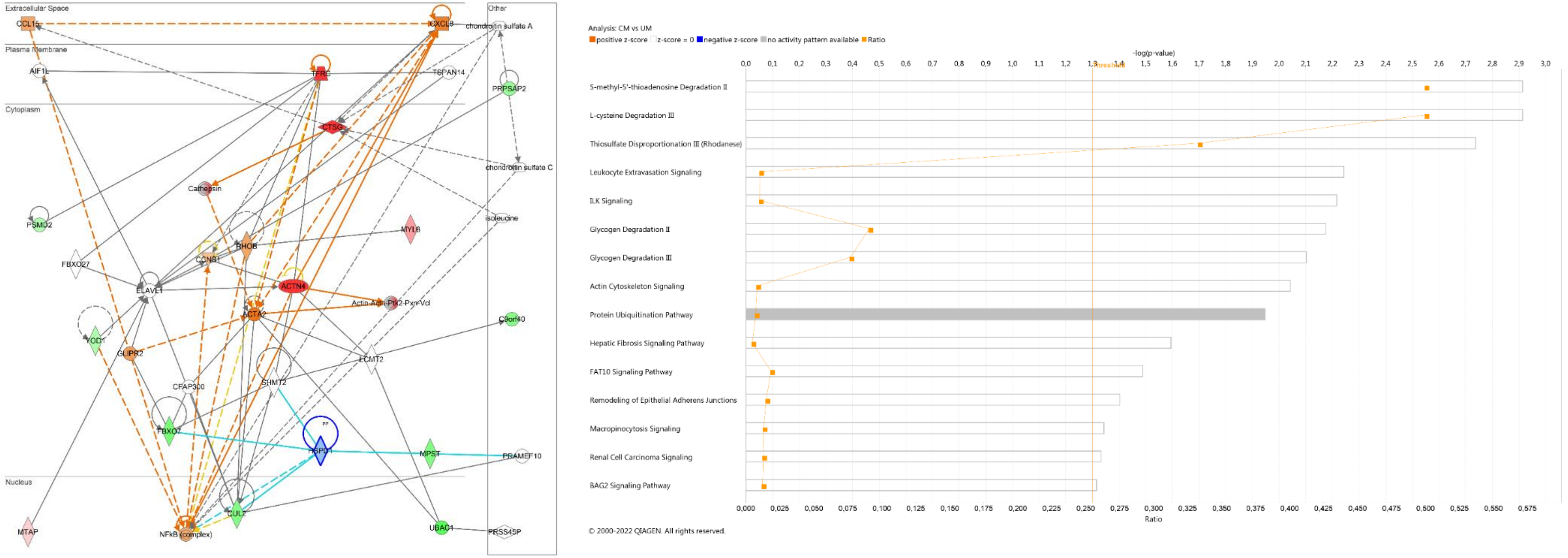

B.

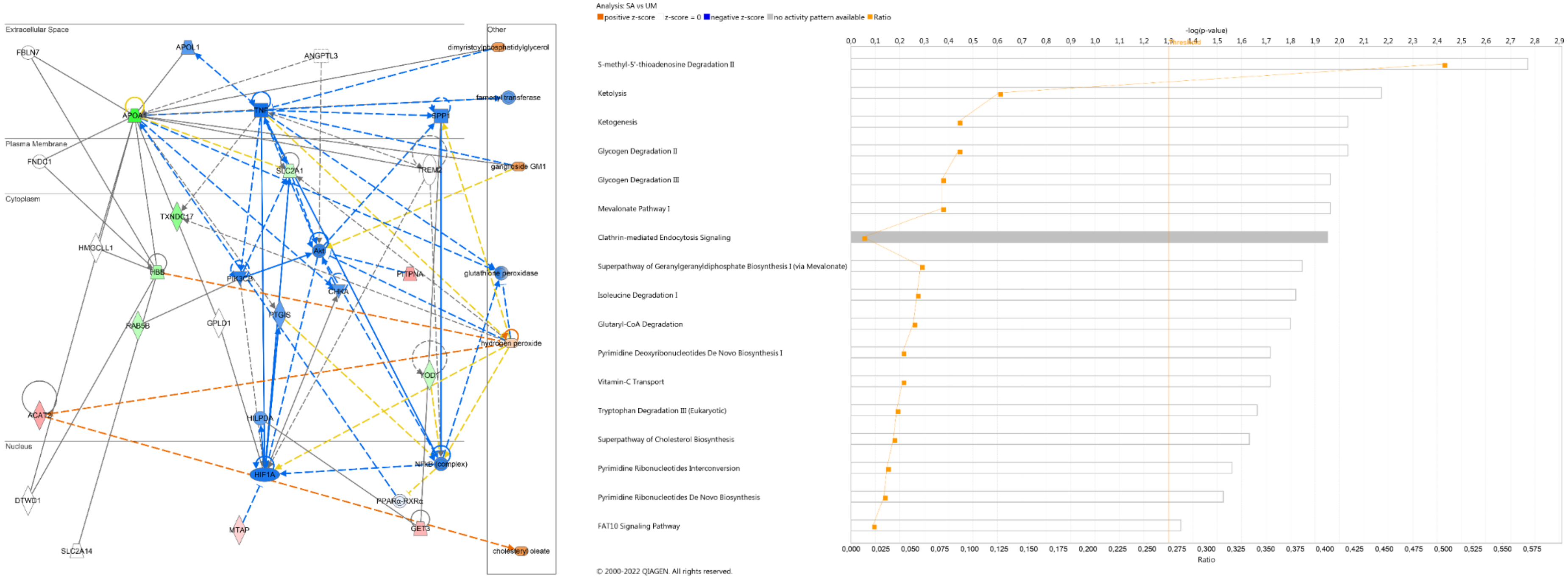

Appendix Figure S2.

C.

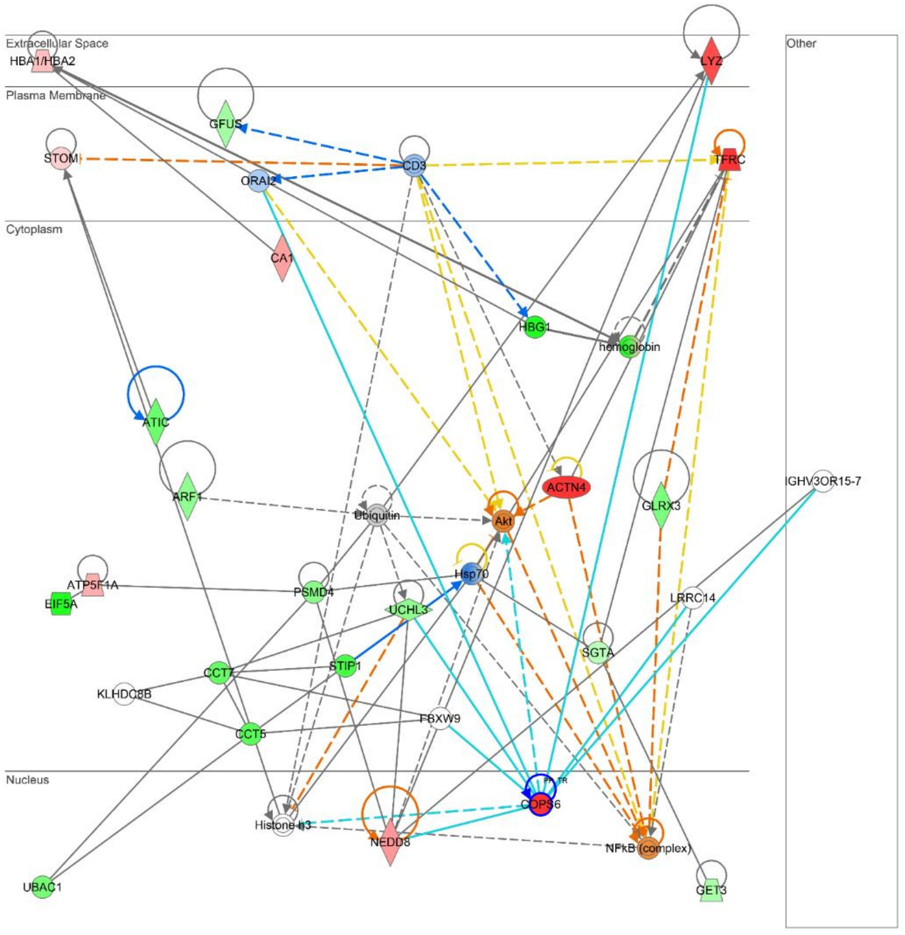

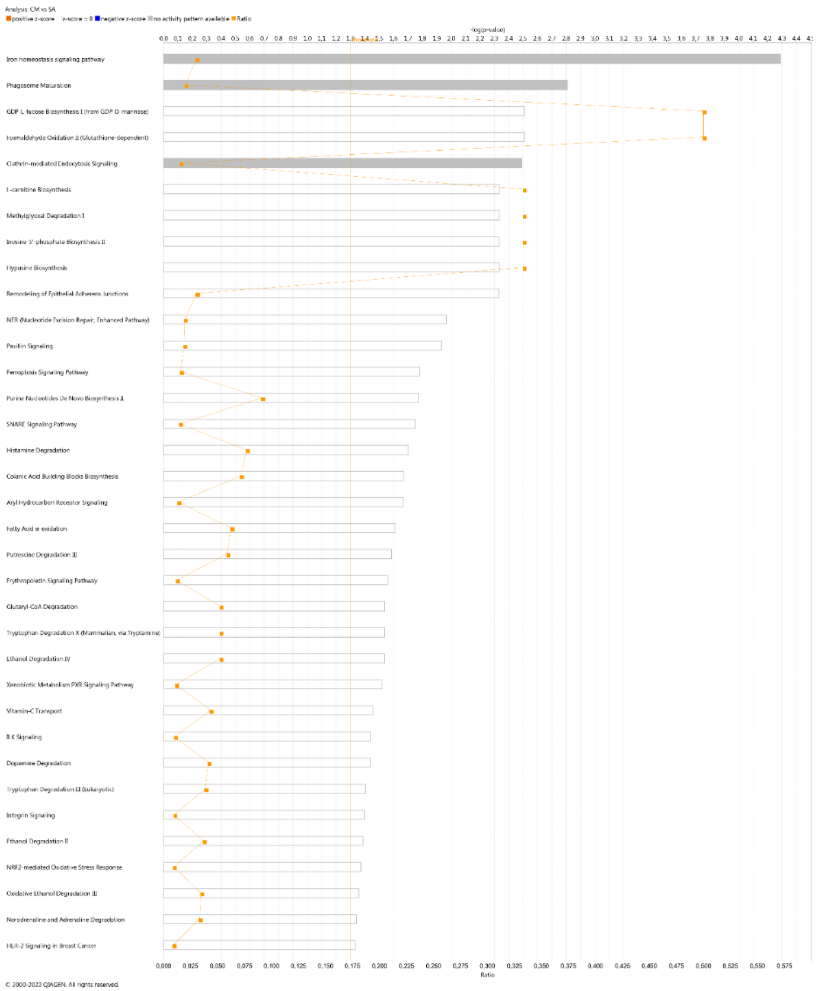

Appendix figure S2. Interaction networks and over-represented pathways from iE differentially abundant Host proteins assessed by Ingenuity pathways Analysis. **A.** CM vs. UM . **B.** UM vs. SMA and **C.** CM vs. SMA

Appendix Figure S3.

A.

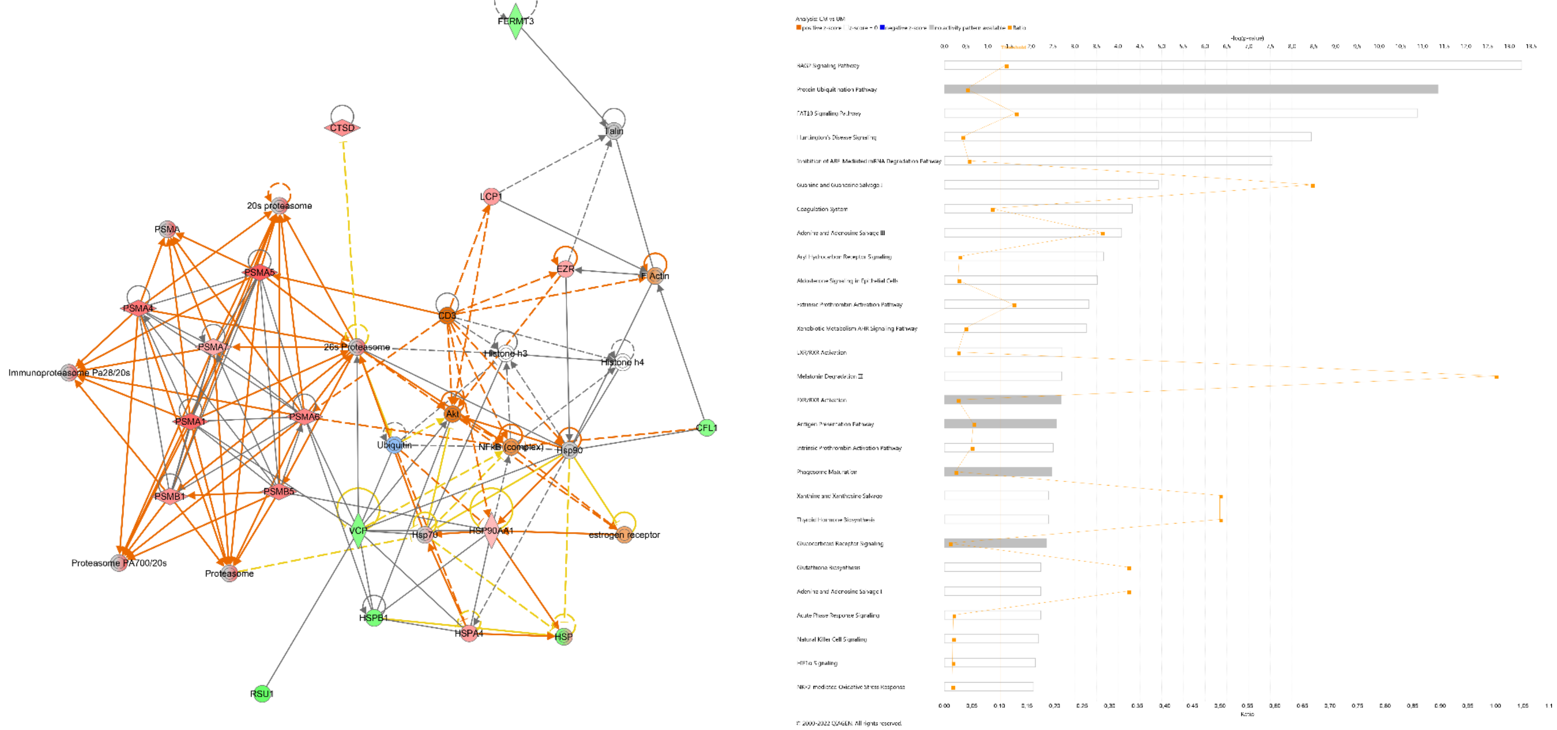

B.

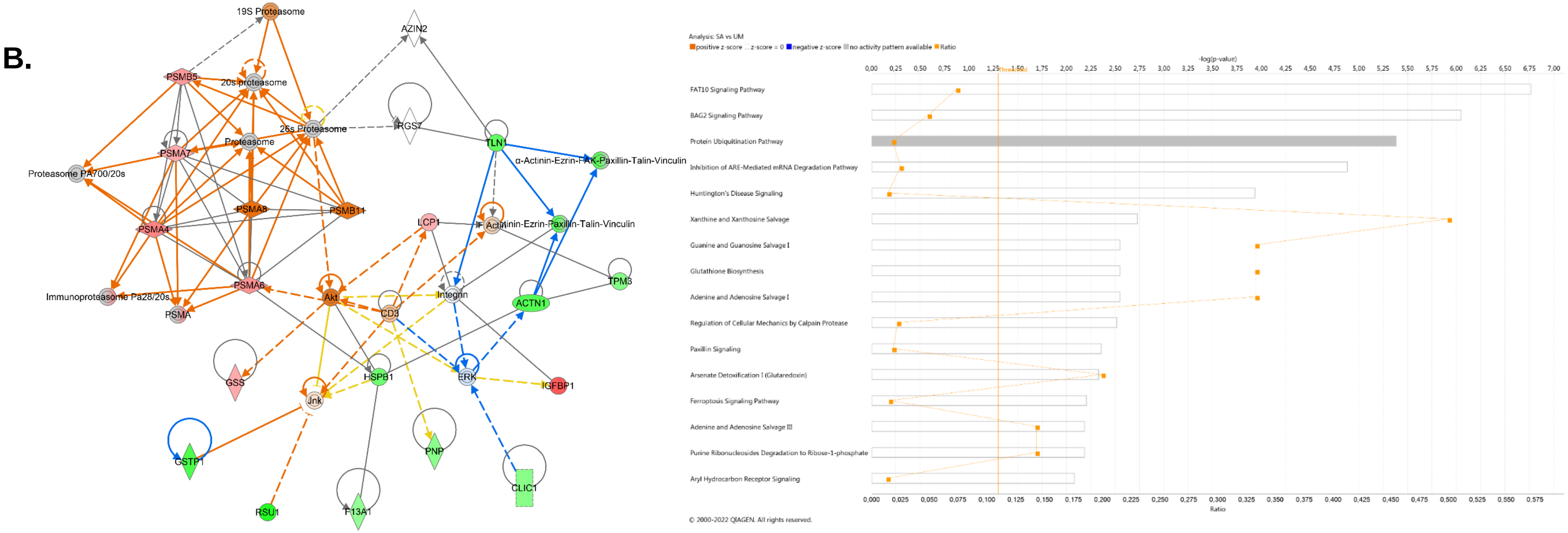

Appendix Figure S3.

C.

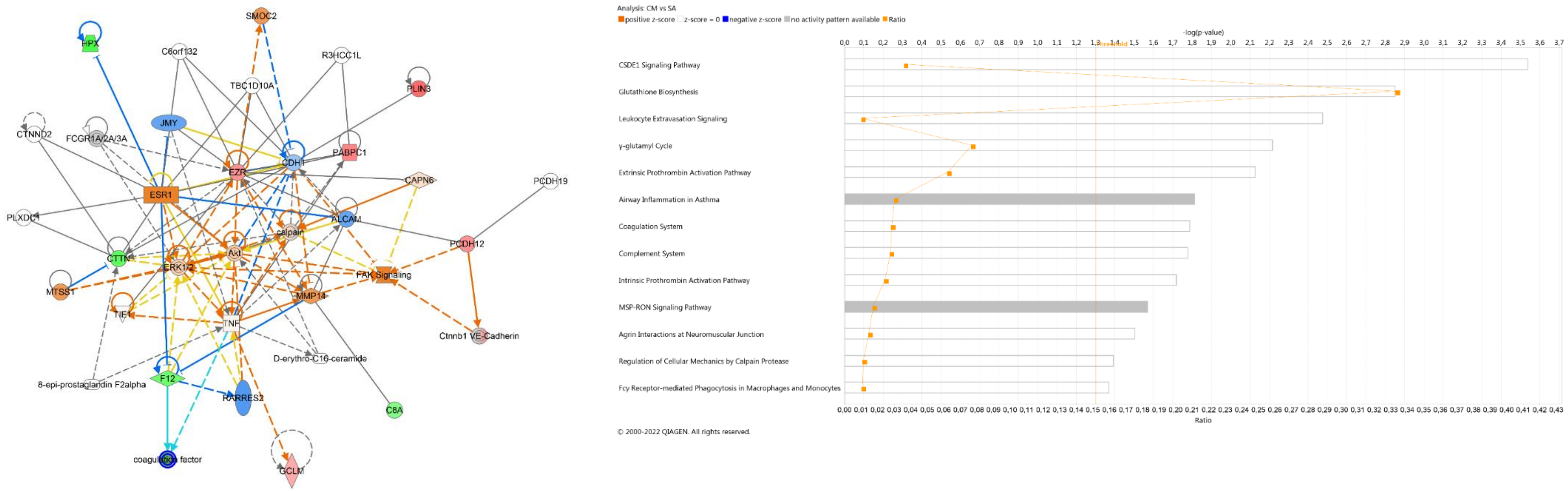

Appendix figure S3. Interaction networks and over-represented pathways from plasma differentially abundant Host proteins assessed by Ingenuity pathways Analysis. **A.** CM vs. UM . **B.** UM vs. SMA and **C.** CM vs. SMA

Appendix Figure S4.

A.

PANTHER GO-Slim Biological Process	Plasmodium falciparum (REF) #	upload_1 (▼ Hierarchy NEW! ?) #	expected	Fold Enrichment	+/-	raw P value
polyamine biosynthetic process	1	1	.00	> 100	+	7.49E-03
↳cellular nitrogen compound biosynthetic process	349	5	1.31	3.80	+	8.48E-03
↳cellular process	1590	11	5.99	1.84	+	2.46E-02
↳cellular biosynthetic process	427	6	1.61	3.73	+	4.02E-03
↳biosynthetic process	433	6	1.63	3.68	+	4.30E-03
↳cellular biogenic amine metabolic process	3	1	.01	88.52	+	1.49E-02
↳cellular amine metabolic process	3	1	.01	88.52	+	1.49E-02
↳amine metabolic process	3	1	.01	88.52	+	1.49E-02
↳organonitrogen compound metabolic process	593	6	2.23	2.69	+	1.91E-02
↳organonitrogen compound biosynthetic process	273	4	1.03	3.89	+	1.79E-02
↳organic substance biosynthetic process	432	6	1.63	3.69	+	4.25E-03
aspartate family amino acid biosynthetic process	2	1	.01	> 100	+	1.12E-02
↳alpha-amino acid biosynthetic process	11	1	.04	24.14	+	4.41E-02
↳alpha-amino acid metabolic process	18	2	.07	29.51	+	2.44E-03
↳cellular amino acid metabolic process	59	3	.22	13.50	+	1.48E-03
↳carboxylic acid metabolic process	101	3	.38	7.89	+	6.46E-03
↳oxoacid metabolic process	101	3	.38	7.89	+	6.46E-03
↳organic acid metabolic process	101	3	.38	7.89	+	6.46E-03
↳small molecule metabolic process	172	3	.65	4.63	+	2.63E-02
↳cellular amino acid biosynthetic process	11	1	.04	24.14	+	4.41E-02
↳aspartate family amino acid metabolic process	2	1	.01	> 100	+	1.12E-02
SCF-dependent proteasomal ubiquitin-dependent protein catabolic process	4	1	.02	66.39	+	1.86E-02
protein stabilization	4	1	.02	66.39	+	1.86E-02
↳regulation of protein stability	4	1	.02	66.39	+	1.86E-02
mitotic DNA replication initiation	5	1	.02	53.11	+	2.23E-02
↳mitotic DNA replication	6	1	.02	44.26	+	2.60E-02
↳nuclear DNA replication	6	1	.02	44.26	+	2.60E-02
↳cell cycle DNA replication	6	1	.02	44.26	+	2.60E-02
↳cellular macromolecule biosynthetic process	313	4	1.18	3.39	+	2.79E-02
↳macromolecule biosynthetic process	313	4	1.18	3.39	+	2.79E-02
↳DNA replication initiation	12	1	.05	22.13	+	4.77E-02
chaperone cofactor-dependent protein refolding	16	2	.06	33.19	+	1.97E-03
↳chaperone-mediated protein folding	17	2	.06	31.24	+	2.20E-03
↳protein folding	67	3	.25	11.89	+	2.11E-03
↳'de novo' protein folding	18	2	.07	29.51	+	2.44E-03
DNA strand elongation involved in DNA replication	8	1	.03	33.19	+	3.33E-02
double-strand break repair via break-induced replication	9	1	.03	29.51	+	3.69E-02
ribosomal subunit export from nucleus	9	1	.03	29.51	+	3.69E-02
cellular response to heat	10	1	.04	26.55	+	4.05E-02
Unclassified	3673	9	13.83	.65	-	2.75E-02

B.

PANTHER GO-Slim Biological Process	Plasmodium falciparum (REF) #	upload_1 (▼ Hierarchy NEW! ?) #	expected	Fold Enrichment	+/-	raw P value
monocarboxylic acid catabolic process	3	1	.02	50.58	+	2.59E-02
↳organic acid metabolic process	101	4	.67	6.01	+	4.61E-03
↳cellular process	1590	17	10.48	1.62	+	2.48E-02
↳small molecule metabolic process	172	5	1.13	4.41	+	5.45E-03
↳carboxylic acid metabolic process	101	4	.67	6.01	+	4.61E-03
↳oxoacid metabolic process	101	4	.67	6.01	+	4.61E-03
↳monocarboxylic acid metabolic process	40	2	.26	7.59	+	3.04E-02
cellular carbohydrate catabolic process	3	1	.02	50.58	+	2.59E-02
↳cellular carbohydrate metabolic process	6	1	.04	25.29	+	4.50E-02
vacuolar acidification	3	1	.02	50.58	+	2.59E-02
↳intracellular pH reduction	3	1	.02	50.58	+	2.59E-02
↳pH reduction	3	1	.02	50.58	+	2.59E-02
↳regulation of pH	3	1	.02	50.58	+	2.59E-02
↳monovalent inorganic cation homeostasis	3	1	.02	50.58	+	2.59E-02
↳regulation of intracellular pH	3	1	.02	50.58	+	2.59E-02
↳regulation of cellular pH	3	1	.02	50.58	+	2.59E-02
↳cellular monovalent inorganic cation homeostasis	3	1	.02	50.58	+	2.59E-02
glucose 6-phosphate metabolic process	5	1	.03	30.35	+	3.87E-02
NADP metabolic process	5	1	.03	30.35	+	3.87E-02
cytoplasmic translational initiation	10	2	.07	30.35	+	2.64E-03
↳translational initiation	20	3	.13	22.76	+	4.16E-04
↳translation	154	5	1.01	4.93	+	3.45E-03
↳cellular macromolecule biosynthetic process	313	6	2.06	2.91	+	1.61E-02
↳macromolecule biosynthetic process	313	6	2.06	2.91	+	1.61E-02
↳organic substance biosynthetic process	432	7	2.85	2.46	+	2.15E-02
↳biosynthetic process	433	7	2.85	2.45	+	2.17E-02
↳cellular biosynthetic process	427	7	2.81	2.49	+	2.03E-02
↳peptide biosynthetic process	155	5	1.02	4.89	+	3.54E-03
↳peptide metabolic process	163	5	1.07	4.65	+	4.36E-03
↳cellular amide metabolic process	174	5	1.15	4.36	+	5.71E-03
↳amide biosynthetic process	162	5	1.07	4.68	+	4.26E-03
↳cellular nitrogen compound biosynthetic process	349	7	2.30	3.04	+	7.25E-03
↳organonitrogen compound biosynthetic process	273	6	1.80	3.34	+	8.63E-03
↳cytoplasmic translation	31	2	.20	9.79	+	1.94E-02
intra-Golgi vesicle-mediated transport	11	2	.07	27.59	+	3.10E-03
↳Golgi vesicle transport	65	3	.43	7.00	+	9.63E-03
↳transport	289	6	1.90	3.15	+	1.12E-02
↳establishment of localization	293	6	1.93	3.11	+	1.19E-02
↳localization	317	6	2.09	2.87	+	1.70E-02
retrograde transport, endosome to Golgi	6	1	.04	25.29	+	4.50E-02
↳intracellular transport	180	6	1.19	5.06	+	1.14E-03
↳cellular localization	211	6	1.39	4.31	+	2.52E-03
↳establishment of localization in cell	185	6	1.22	4.92	+	1.31E-03
mitotic DNA replication	6	1	.04	25.29	+	4.50E-02
↳nuclear DNA replication	6	1	.04	25.29	+	4.50E-02
↳cell cycle DNA replication	6	1	.04	25.29	+	4.50E-02
generation of precursor metabolites and energy	31	2	.20	9.79	+	1.94E-02
endoplasmic reticulum to Golgi vesicle-mediated transport	34	2	.22	8.93	+	2.28E-02
translational elongation	154	5	1.01	4.93	+	3.45E-03
Unclassified	3673	18	24.21	.74	-	2.80E-02

Appendix Figure S4.

C.

	Plasmodium falciparum (REF)	upload_1 (Hierarchy NEW!)				
PANTHER GO-Slim Biological Process	#	#	expected	Fold Enrichment	+/-	raw P value
positive regulation of transcription elongation from RNA polymerase II promoter	4	1	.02	45.78	+	2.69E-02
↳positive regulation of DNA-templated transcription, elongation	4	1	.02	45.78	+	2.69E-02
↳regulation of DNA-templated transcription, elongation	7	1	.04	26.16	+	4.27E-02
↳regulation of transcription elongation from RNA polymerase II promoter	7	1	.04	26.16	+	4.27E-02
inorganic anion transport	4	1	.02	45.78	+	2.69E-02
↳ion transport	44	2	.24	8.32	+	2.54E-02
DNA replication-independent chromatin organization	4	1	.02	45.78	+	2.69E-02
↳chromatin organization	33	2	.18	11.10	+	1.52E-02
↳cellular component organization	372	5	2.03	2.46	+	4.98E-02
↳chromosome organization	69	3	.38	7.96	+	6.68E-03
↳organelle organization	294	5	1.61	3.11	+	2.09E-02
double-strand break repair via break-induced replication	9	2	.05	40.70	+	1.52E-03
↳double-strand break repair via homologous recombination	27	2	.15	13.57	+	1.06E-02
↳recombinational repair	28	2	.15	13.08	+	1.13E-02
↳DNA recombination	38	2	.21	9.64	+	1.96E-02
↳double-strand break repair	33	2	.18	11.10	+	1.52E-02
mitotic DNA replication initiation	5	1	.03	36.63	+	3.22E-02
↳mitotic DNA replication	6	2	.03	61.05	+	7.81E-04
↳nuclear DNA replication	6	2	.03	61.05	+	7.81E-04
↳cell cycle DNA replication	6	2	.03	61.05	+	7.81E-04
↳DNA-dependent DNA replication	35	2	.19	10.47	+	1.69E-02
↳DNA replication	38	2	.21	9.64	+	1.96E-02
↳organic substance biosynthetic process	432	6	2.36	2.54	+	2.76E-02
↳biosynthetic process	433	6	2.36	2.54	+	2.79E-02
↳cellular biosynthetic process	427	6	2.33	2.57	+	2.62E-02
↳mitotic cell cycle process	59	2	.32	6.21	+	4.28E-02
↳mitotic cell cycle	59	2	.32	6.21	+	4.28E-02
nucleosome assembly	6	1	.03	30.52	+	3.74E-02
↳DNA conformation change	28	2	.15	13.08	+	1.13E-02
DNA unwinding involved in DNA replication	7	1	.04	26.16	+	4.27E-02
COPII-coated vesicle budding	7	1	.04	26.16	+	4.27E-02
↳endoplasmic reticulum to Golgi vesicle-mediated transport	34	2	.19	10.77	+	1.60E-02
DNA strand elongation involved in DNA replication	8	1	.04	22.89	+	4.79E-02
nucleotide biosynthetic process	38	2	.21	9.64	+	1.96E-02
↳nucleoside phosphate biosynthetic process	38	2	.21	9.64	+	1.96E-02
↳nucleobase-containing compound biosynthetic process	171	5	.93	5.35	+	2.29E-03
↳organic cyclic compound biosynthetic process	192	5	1.05	4.77	+	3.74E-03
↳cellular nitrogen compound biosynthetic process	349	6	1.91	3.15	+	1.06E-02
↳aromatic compound biosynthetic process	190	5	1.04	4.82	+	3.58E-03
↳heterocycle biosynthetic process	191	5	1.04	4.79	+	3.66E-03
↳nucleoside phosphate metabolic process	53	2	.29	6.91	+	3.54E-02
↳nucleobase-containing small molecule metabolic process	63	2	.34	5.81	+	4.80E-02
↳nucleotide metabolic process	50	2	.27	7.33	+	3.19E-02
DNA biosynthetic process	42	2	.23	8.72	+	2.34E-02
mitotic nuclear division	59	2	.32	6.21	+	4.28E-02

Appendix figure S4. Over-represented biological pathways from iE differentially abundant parasitic proteins assessed by PANTHER GO online tool. A. CM vs. UM . **B.** UM vs. SMA and **C.** CM vs. SMA DAP.

Appendix Figure S5.

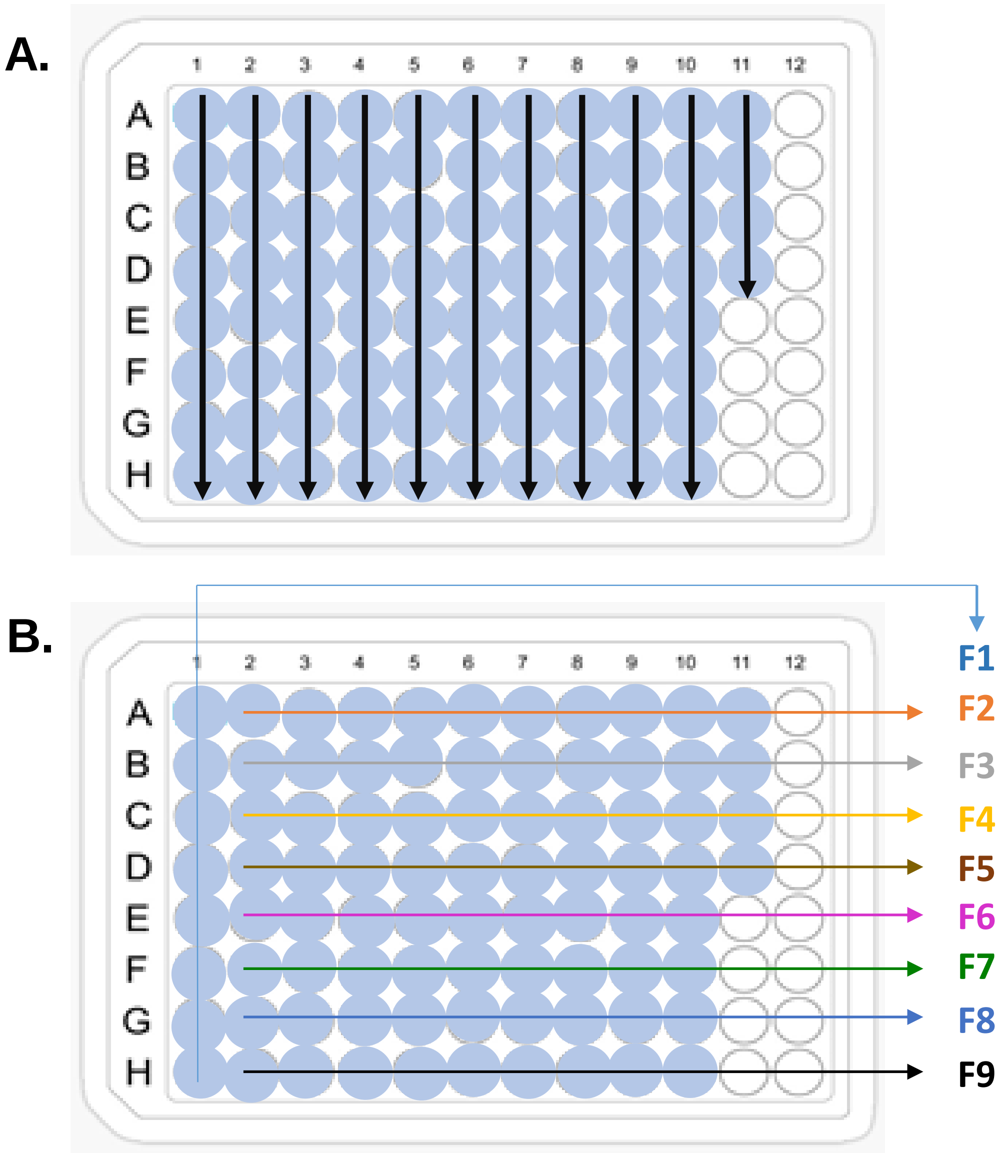

Appendix figure S5. Reverse-Phase High pH Chromatography fractionation. A. AKTA HpH chromatography sampling collection. **B.** Concatenation scheme of the 84 fractions obtained after HpH chromatography. F1 is considered as a flow through but was injected.
