# Supplementary material for: Infected erythrocytes and plasma proteomics reveal a specific protein signature of severe malaria
Source: EMBO Mol Med. 2024 Jan 31;16(2):6. doi: 10.1038/s44321-023-00010-0 (PMC10897182; doi:10.1038/s44321-023-00010-0)
Supplement: Supplementary file 16 — Expanded View Figures [file 44321_2023_10_MOESM16_ESM.pdf]

# Expanded View Figures

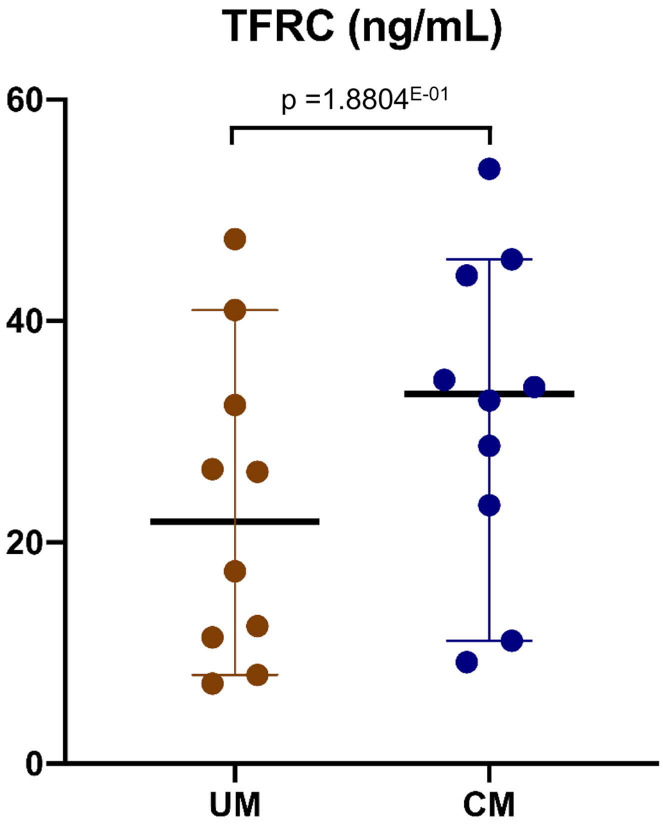


**Figure EV1. Targeted quantification of transferrin receptor protein 1 (*TFRC*) by ELISA assay.**

Dot plot representing *TFRC* concentration of 20 iE samples from the NeuroCM cohort (10 UM vs 10 CM) measured by ELISA assay. Data were analyzed with GraphPad Prism 8.0. Bold horizontal bars correspond to the median and colored bars correspond to the 95% CI. Mann–Whitney *U*-test was used as statistical test and *p* value is displayed in the figure. Source data are available online for this figure.

© This is a U.S. Government work and not under copyright protection in the US; foreign copyright protection may apply
